# Prevalence and predictors of maternal smoking prior to and during pregnancy in a regional Danish population: a cross-sectional study

**DOI:** 10.1186/s12978-019-0740-7

**Published:** 2019-06-14

**Authors:** Mie Gaarskjaer de Wolff, Mette Grønbæk Backhausen, Mette Langeland Iversen, Jane Marie Bendix, Ane Lilleøre Rom, Hanne Kristine Hegaard

**Affiliations:** 1 Department of Obstetrics, Copenhagen University Hospital, Rigshospitalet, Copenhagen, Denmark; 2 Research Unit Women’s and Children’s Health, The Juliane Marie Centre, Copenhagen University Hospital, Rigshospitalet, Blegdamsvej 9, 2100 Copenhagen, Denmark; 3 Department of Gynecology and Obstetrics, Zealand University Hospital, Sygehusvej 10, 4000 Roskilde, Denmark; 4 Department of Gynecology and Obstetrics, Nordsjællands Hospital, Hillerød, University of Copenhagen, Dyrehavevej 29, 3400 Hillerød, Denmark; 5 The Institute of Clinical Medicine, Faculty of Health and Medical Sciences, University of Copenhagen, Blegdamsvej 3, Copenhagen, Denmark

**Keywords:** Maternal smoking, Pregnancy, Socioeconomic status, Antenatal care, Health inequality

## Abstract

**Background:**

Maternal smoking is still a major public health problem posing the risk of several negative health outcomes for both the pregnant woman and her offspring. The prevalence of maternal smoking in Denmark and other high-income countries has decreased continuously since the 1980s, and a prevalence below 10% of women who continue to smoke during pregnancy has been reported in studies after 2010. Previous studies have shown that low socioeconomic status is associated with maternal smoking. Information from the Danish Birth Register about maternal smoking shows that the prevalence of women who report to smoke in pregnancy has decreased continuously with 23.3% who reported ever smoking in pregnancy in 2000, 12.9% in 2010 and 9.0% in 2017. The aim of this study was to estimate the prevalence of maternal smoking at the time of conception and at 20 weeks of gestation in a regional Danish population, to describe differences in maternal characteristics among smokers, quitters and never-smokers, and to estimate predictors of smoking at the time of conception.

**Methods:**

A cross-sectional study was conducted among pregnant women receiving antenatal care at the Department of Obstetrics, Zealand University Hospital, Denmark from August 2015 to March 2016 (*n* = 566). The main outcome was smoking at the time of conception and at 20 weeks of gestation. The questionnaire also collected information about maternal, health-related and sociodemographic characteristics. Descriptive analysis was conducted, and multivariate logistic regression analysis was used to assess the potential associated predictors (adjusted odds ratio).

**Results:**

The prevalence of self-reported smoking at the time of conception was 16% (*n* = 90) and 6% smoked at 20 weeks of gestation (*n* = 35), as 61% of smokers quit smoking during early pregnancy. Multiple logistic regression analysis showed that significant predictors for smoking at conception were the socioeconomic factors; ≤12 years of education, shift work and being unemployed.

**Conclusion:**

The prevalence of self-reported maternal smoking in this regional Danish population of pregnant women is lower than seen in previous studies. However, predictors for smoking at the time of conception remain to be factors of low socioeconomic status confirming a social inequality in maternal smoking. Women at risk of smoking during pregnancy must be identified in early pregnancy or even before pregnancy and be offered interventions to help them quit smoking.

## Plain English summary

During the past decades the number of pregnant smokers as well as smokers in the general population, has decreased in Denmark. The public awareness has shifted, and smoking has been prohibited in public places such as work places and bars. Also, the growing knowledge about the negative health effects of smoking has become widespread in the population, and the attitude towards maternal smoking from the surroundings has become increasingly negative.

However, smoking during pregnancy is still a major public health concern as recent studies from other high-income countries have shown that, despite the decrease, a proportion between 5 and 15% of all pregnant women continues to smoke during pregnancy, leaving themselves and their offspring at risk of major negative health outcomes such as preterm birth, birth defects and low birth weight. The aim of this study was to estimate the prevalence of self-reported smoking in a Danish population and to identify possible risk factors for smoking in pregnancy.

We conducted a questionnaire study at the Department of Obstetrics, Zeeland University Hospital in Denmark from August 2015 – March 2016 among 566 women who attended antenatal care at the hospital. The questionnaire contained information regarding smoking habits at the time of conception and at 20 weeks of pregnancy and questions about maternal characteristics, health, and socioeconomic characteristics.

The results of our study were in accordance with other recent studies from high-income countries. The prevalence of smoking at the time of conception was 16% and this prevalence decreased to 6% during pregnancy. We found that the women with low socioeconomic status had a significantly higher risk of smoking at the time of conception and were more likely to continue smoking during pregnancy.

## Background

Maternal smoking is one of the most harmful risk factors during pregnancy, potentially leading to adverse pregnancy outcomes and negative impacts on the offspring [1]. Smoking during pregnancy significantly increases the risk of spontaneous abortion [2], placental abruption [3], preterm birth [4], still birth [5], low birth weight [6], neonatal infections [7], and specific congenital malformations, including malformations in the cardiovascular system and digestive system [8]. Furthermore, smoking has been associated with negative long-term health outcomes in the offspring, including impaired intellectual development, learning deficits [9, 10], as well as reduced sperm quality in the male offspring [11]. Smoking cessation during pregnancy has been found to reduce or even to eliminate the risk of adverse pregnancy outcomes together with the negative long-term health outcomes in the offspring [12–14].

In line with health authorities in many other countries, the Danish National Health Authorities have advised women for decades to abstain from smoking during pregnancy and have offered pregnant smokers interventions to help them quit smoking [15–17]. Furthermore, in Denmark legal acts have been implemented to decrease the prevalence of active and passive smoking; since 2007, smoking has not been allowed in Danish workplaces or public areas, including restaurants and bars, [18].

The prevalence of smoking during pregnancy has decreased in high-income countries, from 20 to 35% in the 80s–90s [19] to 10–20% in the 00s [5, 20–22] and even a prevalence below 10% has been reported in studies after 2010 [23–26]. In Denmark, studies based on data from the Danish National Birth Register (DNBR) have shown a similar decrease in prevalence [5, 27] and women who reported ever-smoking in pregnancy was 23.1% in 2000 and 12.9% in 2010. The latest data from 2017 in DNBR show that 9.0% of the women were ever-smokers in pregnancy while 6.7% continued to smoke during pregnancy [28].

Maternal smoking is a marker of social inequality [29], with higher rates of pregnant smokers observed among women with lower educational level, lower income, and among women living in socially deprived areas [22, 30]. Further employment status has been found to be associated with smoking in pregnancy [31]. Among non-pregnant women shift work has been recognized as a predictor of smoking [32, 33] and to our knowledge this has not been investigated among pregnant women. Finally, previous studies have found that smoking cessation success rates during pregnancy were significantly higher for women of higher socioeconomic status [29, 30, 34].

In order to develop targeted interventions that support women in smoking cessation before or during pregnancy more knowledge is needed about the characteristics of the women who quit smoking and of those who continue to smoke during pregnancy. Further there is a need to investigate if the observed decrease in maternal smoking is a continuous. Moreover, whether the social inequality in maternal smoking is becoming increasingly strong as it has been observed in non-pregnant female populations since 2000 [35].

Hence the aim of this study was to estimate the prevalence of maternal smoking at the time of conception and at 20 weeks of gestation in a regional Danish population, to describe differences in maternal characteristics among smokers, quitters and never-smokers and to estimate predictors of smoking at the time of conception.

## Methods

In the current cross-sectional study, we used data from the Low Back Pain Study [36], which was conducted at the Department of Obstetrics, Zealand University Hospital, Denmark from August 2015 to March 2016. The hospital is one of four hospitals providing obstetric services in the Region of Zealand and has approximately 2600 deliveries annually.

Pregnant women aged ≥18 years who were able to understand and speak Danish were invited to participate in the study when they attended an appointment for a routine ultrasound scan at 20 weeks of gestation. Approximately 95% of all pregnant women receive this ultrasound scan [37], which is a standard part of the free public antenatal care in Denmark.

In total, 786 women attended the ultrasound scan appointment during the study period. We excluded 96 women because they did not understand or speak Danish, and 36 women were not approached due to logistical failures, leaving a total of 654 eligible women. All eligible women were asked to complete an electronic questionnaire immediately after the routine ultrasound scan. A total of 566 (87%) completed the questionnaire and comprised the final study population.

Our outcome variable was self-reported smoking status, which was gathered using the following questions: *Did you smoke at the time of conception? (yes/no).* Followed by: *If yes, how many cigarettes did you smoke daily?* (number of cigarettes smoked daily). The question concerning smoking at 20 weeks of gestation was phrased identically: *Do you smoke currently? (yes/no)*. Followed by: *If yes, how many cigarettes do you smoke daily?* (number of cigarettes smoked daily). These questions have been used in previous smoking studies [23, 38]. The outcome variable was categorized into the following: smoking status at the time of conception and at 20 weeks of gestation (yes/no). The number of cigarettes per day were categorized as 1–9 cigarettes per day and ≥ 10 cigarettes per day, as reported in previous studies [38, 39].

The questionnaire also collected information about: 1) maternal characteristics: *age*, *pre-pregnancy Body Mass Index* (BMI) kg/m^2^, *parity*, and *mode of conception* (spontaneous or assisted reproductive technologies (ART)); 2) health-related characteristics: *current diagnosis of chronic illness* (somatic or mental diseases chosen from a predefined list: hypertension, lung disease, diabetes type 1 and 2, metabolic disease, kidney disease, epilepsy, arthritis, heart disease, and not-specified mental disease), and *self-rated general health* measured by one item from the 36-item short-form survey instrument [40] containing five response options (excellent, very good, good, fair and poor) which were analyzed as three categories (excellent and very good as good, good and fair as moderate, and poor as poor); and 3) socioeconomic characteristics: *level of education* (compulsory = 9 years mandatory schooling or skilled worker = 9 years of mandatory schooling and 3 as a trainee (categorized together as ≤12 years of education), and 1–4 years higher education or advanced academic degree (categorized as > 12 years of education)), *work schedule* (daytime (working in normal daytime) or shift work (defined as working evening and/or night hours), *employment status* (employed or unemployed), and *cohabitation* (yes/no).

### Statistical analysis

Descriptive statistics were applied to calculate mean values with standard deviations and percentages. Pearson’s chi-square test and Fischer’s Exact Test was used to determine significance between groups of categorical variables, and for continuous variables the Student’s t-test or one-way ANOVA was applied.

To examine associations between potential predictors and smoking at the time of conception, univariate and multiple logistic regression analyses were performed and presented as crude odds ratios (OR) and adjusted odds ratios (aOR) with 95% confidence intervals (CI). Logistic regression analysis was not performed for smoking at 20 weeks of gestations due to the small number of pregnant smokers (*n* = 35) at this time point.

Maternal age, parity, cohabitation, level of education, and employment status were a priori considered potential predictors [20, 26, 41]. Furthermore, a variable work schedule (daytime or shift work) was included in the regression model, as we hypothesized that work schedule could also be a potential predictor of maternal smoking. All potential predictors were categorized as shown in Table 1.

**Table 1:** Characteristics of the study population (*n* = 566) at the time of conception and at 20 weeks of gestation

	At the time of conception				At 20 weeks of gestation			
	Total	Smokers	Never smokers	*P*-value*	Smokers	Quitters	Never smokers	*P*-value*
	n	n (%)	n (%)		n (%)	n (%)	n (%)	
Overall respondents	566	90 (16)	476 (84)		35 (6)	55 (10)	476 (84)	
Age (years)				0.002				0.01
18–24	55	17 (31)	38 (69)		7 (13)	10 (18)	38 (69)	
25–29	184	35 (19)	149 (81)		12 (7)	23 (12)	149 (81)	
30–34	205	25 (12)	180 (88)		9 (4)	16 (8)	180 (88)	
≥ 35	122	13 (11)	109 (89)		7 (6)	6(5)	109 (89)	
Mean age (SD)	30.5	29 (5.2)	30.8 (4.8)	0.002	29.7	28.6	30.8 (4.8)	0.004
Missing data 0								
BMI (kg/m^2^)				0.015				0.31
Underweight (< 18.5)	19	8 (42)	11 (58)		3 (16)	5 (26)	11 (58)	
Normal (18.5–24.9)	302	45 (15)	257 (85)		17 (6)	28 (9)	257 (85)	
Overweight (25–29.9)	134	22 (16)	112 (84)		11 (8)	11 (8)	112 (84)	
Obese (≥ 30)	64	8 (12)	56 (88)		4 (6)	4 (6)	56 (88)	
Mean BMI (SD)	24.4	24.8 (4.5)	24.3 (4.7)		25.0 (4.7)	24.6 (4.5)	24.3 (4.7)	0.33
Missing data 47								
Parity				0.04				0.02
Nullipara	222	44 (20)	178 (80)		13 (6)	31(14.0)	178 (80)	
Multipara	343	46 (13)	297 (87)		22 (6)	24 (7)	297 (87)	
Missing data 1								
Mode of conception				0.21				0.29
Spontaneous	504	83 (16)	421 (84)		34 (7)	49 (10)	421 (83)	
Assisted reproductive technologies	59	6 (10)	53 (90)		1 (2)	5 (8)	53 (90)	
Missing data 3								
Chronic illness^a^				0.90				0.94
Yes	104	17 (16)	87 (84)		6 (6)	11 (10)	87 (84)	
No	462	73 (16)	389 (84)		29 (6)	44 (10)	389 (84)	
Missing data 0								
Self-rated general health				0.11				0.14
Good	411	62 (15)	349 (85)		21 (5)	41 (10)	349 (85)	
Moderate	119	18 (15)	101 (85)		9 (8)	9(8)	101 (84)	
Poor	35	10 (29)	25 (71)		5 (14)	5(14)	25 (72)	
Missing data 1								
Education				< 0.001				< 0.0001
≤ 12 years	101	30 (30)	71 (70)		17 (17)	13 (13)	71 (70)	
> 12 years	463	60 (13)	403 (87)		18 (4)	42 (9)	403 (87)	
Missing data 2								
Work schedule				0.001				0.002
Day time	465	63 (13)	402 (87)		22 (5)	41 (9)	402 (86)	
Shift work	101	27 (27)	74 (73)		13 (13)	14 (14)	74 (73)	
Missing data 0								
Employment status				< 0.001				< 0.0001
Employed	509	71 (14)	438 (86)		24 (5)	47 (9)	438 (86)	
Unemployed	57	19 (33)	38 (67)		11 (19)	8 (14)	38 (67)	
Missing data 0								
Cohabitation				0.008				< 0.0001
Yes	526	77 (15)	449 (85)		26 (5)	51 (10)	449 (85)	
No	39	12 (31)	27 (69)		8 (21)	4 (10)	27 (69)	
Missing data 1								
Cigarettes per day^b^								
1–9 cigarettes	N/A	24 (29)	N/A	–		N/A	N/A	–
≥10 cigarettes	N/A	60 (71)	N/A	–		N/A	N/A	–
Missing data 6								

*N/A* Not Applicable

* *P*-values for Pearson Chi-square test or if less than 5 expected cell count p-value for Fischer’s Exact Test. For continuous variables Student’s T test or ANOVA were used

^a^Hypertension, lung disease, diabetes type 1 + 2, metabolic disease, kidney disease, epilepsy, arthritis and heart disease, and not-specified mental disease

^b^ Cigarettes per day are only listed among smokers

The potential predictors were entered in the multiple logistic regression models and were mutually adjusted. A two-sided *p-*value < 0.05 was considered statistically significant. The statistical analyses were performed in SPSS 22.0 (IBM).

## Results

The overall prevalence of reported smoking was 16% (*n* = 90) at the time of conception, among whom 29% reported smoking 1–9 cigarettes per day and 71% ≥10 cigarettes per day. At 20 weeks of gestation, 6% (*n* = 35) reported continued smoking, of whom 77% reported smoking 1–9 cigarettes per day and 23% reported smoking ≥10 cigarettes per day.

Table 1 shows the distribution of maternal characteristics in relation to smoking status in the study population. The highest proportion of smoking at conception was seen in the categories: pre-pregnancy BMI < 18 kg/m^2^ (42%), unemployed (33%), 18–24 years of age (31%), women living alone (31%), ≤12 years of education (30%), poor self-rated general health (29%), and shift work (27%). At 20 weeks of gestation, the highest prevalence of smokers was seen in the categories: women living alone (20%), unemployed (19%), ≤12 years of education (17%), pre-pregnancy BMI < 18 kg/m^2^ (16%), poor self-rated general health (14%), 18–24 years of age (13%), and shift work (13%).

At the time of conception, the proportion of smokers and non-smokers differed statistically significantly within the following maternal characteristics: age, pre-pregnancy BMI, parity, education, work schedule, employment status, and cohabitation. At 20 weeks of gestation, the proportion of smokers, quitters and never-smokers differed statistical significantly within the maternal characteristics: age, parity, education, work schedule, employment status, and cohabitation (Table 1).

Overall, 61% of the women who reported smoking at conception quit smoking during early pregnancy. The highest percentages of quitters were seen among women who smoked 1–9 cigarettes per day at time of conception (88%), women using assisted reproductive technologies (83%), nulliparas (71%), women with > 12 years of education (70%), employed women (66%), cohabiting women (66%), and women working daytime (65%) (Table 2).

**Table 2:** Characteristics of women who quit smoking during pregnancy (*n* = 55) among smokers at conception (n = 90)

	Smokers at conception	Quitters during pregnancy
	n	n (%)
Overall	90	55 (61%)
Age (years)		
18–24	17	10 (59%)
25–29	35	23 (66%)
30–34	25	16 (64%)
≥ 35	13	6 (46%)
Missing data 0		
BMI (kg/m^2^)		
Underweight (< 18.5)	8	5 (63%)
Normal (18.5–24.9)	45	28 (62%)
Overweight (25–29.9)	22	11 (50%)
Obese (≥ 30)	8	4 (50%)
Missing data 7		
Parity		
Nullipara	44	31 (71%)
Multipara	46	24 (52%)
Missing data 0		
Mode of conception		
Spontaneous	83	49 (59%)
Assisted reproductive technologies	6	5 (83%)
Missing data 1		
Chronic illness^a^		
Yes	17	11 (65%)
No	73	44 (62%)
Missing data 0		
Self-rated general health		
Good	62	21 (66%)
Moderate	18	9 (50%)
Poor	10	5 (50%)
Missing data 1		
Education		
≤ 12 years	30	13 (43%)
> 12 years	60	42 (70%)
Missing data 0		
Work schedule		
Day time	63	41 (65%)
Shift work	27	14 (52%)
Missing data 0		
Employment status		
Employed	71	47 (66%)
Unemployed	19	8 (42%)
Missing data 0		
Cohabitation		
Yes	77	51 (66%)
No	12	4 (33%)
Missing data 0		
Cigarettes per day		
1–9 cigarettes	24	21 (88%)
≥ 10 cigarettes	60	31 (52%)
Missing data 6		

^a^Hypertension, lung disease, diabetes type 1 + 2, metabolic disease, kidney disease, epilepsy, arthritis, heart disease, and not-specified mental disease

Predictors for smoking at the time of conception were identified as ≤12 years of school (aOR 2.2 (95% CI: 1.2–3.8)), shift work (aOR 2.6 (95% CI: 1.5–4.6)), and unemployment (aOR 3.2 (95% CI: 1.6–6.2)) (Table 3).

**Table 3:** Logistic regression model with associations of smoking at the time of conception

Potential predictors	At the time of conception			
	Smokers	Non-smokers	Crude OR	Adjusted OR^a^
	n	n	OR (95% CI)	aOR (95% CI)
Overall respondents	90	476		
Age				
18–24	17	38	1.9 (1.0–3.8)	1.2 (0.6–2.5)
25–29	35	149	Ref	Ref.
30–34	25	180	0.6 (0.3–1.0)	0.6 (0.3–1.1)
≥ 35	13	109	0.5 (0.3–1.0)	0.5 (0.2–1.0)
Missing data 0				
Parity				
Nullipara	44	178	Ref.	Ref.
Multipara	46	297	0.6 (0.4–1.0)	0.8 (0.5–1.3)
Missing data 1				
Education				
≤ 12 years	60	403	Ref.	Ref.
> 12 years	30	71	2.8 (1.7–4.7)	2.2 (1.2–3.8)
Missing data 2				
Work schedule				
Day time	63	402	Ref.	Ref.
Shift work	27	74	2.3 (1.4–3.9)	2.6 (1.5–4.6)
Missing data 0				
Employment status				
Employed	71	438	Ref.	Ref.
Unemployed	19	38	3.1 (1.7–5.6)	3.2 (1.6–6.2)
Missing data 0				
Cohabitation				
Yes	77	449	Ref.	Ref.
No	12	27	2.6 (1.3–5.3)	1.7 (0.7–3.8)
Missing data 1				

^a^ In the adjusted model all results are adjusted for age, parity, education, parity, work schedule, occupation, and cohabitation

Furthermore, at the time of conception, we identified a non-significant decreased risk of smoking among older women aged 35 years or older (aOR 0.5 (95% CI: 0.2–1.0)).

Sub-analyses also included pre-pregnancy Body Mass Index (BMI) kg/m^2^ as a potential predictor of smoking during pregnancy. However, these analyses showed essentially unchanged results and were, due to missing data in this category (*n* = 47), not included in the final analysis (data not shown).

## Discussion

The overall prevalence of smoking was 16% at the time of conception and decreased to 6% at 20 weeks of gestation. Significant predictors for smoking at the time of conception were the following socioeconomic factors: ≤12 years of education, unemployment, and shift work.

The estimated prevalence of smoking during pregnancy (6%) was lower than in a previous Danish study from 2005, where the prevalence during pregnancy was 16%. This may in part reflect the decrease in tobacco use seen in the general population in the same period [42]. Our findings are in line with studies from Norway, Canada, and Iceland [20, 24, 43], in which similar decreases in the prevalence of maternal smoking have been found, ranging from 12 to 22% before pregnancy to 5–10% during pregnancy in the same period of time [20, 24, 43].

This present study reported a higher prevalence of smoking among women with lower socioeconomic status at both time points during the study period. Moreover, three of the socioeconomic factors examined (education, employment status, and work schedule) were significant predictors for smoking. These findings are supported by results from other studies [20–23, 29], and suggest that there is still a high degree of social inequality in rates of maternal smoking cessation. Further it emphasizes the need to focus on such vulnerable groups when targeting future smoking cessation interventions among pregnant women and among young women who are planning to become pregnant.

In this study the work-related socioeconomic variables shift work and employment status were associated with an increased risk of smoking at the time of conception and smokers were more likely to be unemployed and to have shift work at 20 weeks of gestation. This is in line with previous studies including data from the American “Nurses studies” [32, 33], showing the same association between shift work and an increased risk of smoking in a non-pregnant population. Employment status has also previously been shown to be a predictor for smoking before and during pregnancy [31]. These finding suggests that an increased clinical focus should be directed towards work-related factors for women prior to and during pregnancy.

Multiparous women and women who smoked ≥10 cigarettes per day were less likely to quit smoking during pregnancy, which also has been found in other studies [29, 34]. The latter confirms the strong biological association found between nicotine dependency and continued smoking in pregnancy [44] [21–23] and could be an explanation for continued smoking during pregnancy.

In the present study 61% of the women who smoked at conception quitted smoking during pregnancy, and the women who continued to smoke reported that they cut down the number of cigarettes per day. Findings from a review of qualitative studies indicate that many pregnant women who smoke perceive a cut down in the number of cigarettes smoked per day as an acceptable method to achieve the goal of quitting smoking and as a means to practice harm reduction [45]. To achieve the goal of total smoking cessation [45], this practice is to some extend acknowledged by midwives and other health providers. However, it is of utmost importance that all pregnant women who smoke, even if they cut down the numbers, are offered continuous intervention to support them in achieving total cessation during pregnancy. A Cochrane review (2017) [34] showed that compared to usual care psychosocial interventions supporting pregnant women in smoking cessation improved the success rate of cessation. Furthermore, the review showed that cessation even late in pregnancy reduced the negative health effects associated with smoking such as low birth weight and stillbirth significantly [5, 34]. These findings highlight the importance of implementing interventions to help pregnant smokers quit smoking as part of the free antenatal care in Denmark and in other countries.

### Strengths and limitations

The sample size is a limitation to the present study. Due to the small number of cases at 20 weeks no logistic regression analysis was performed to identify predictors at 20 weeks. To further investigate the association between for example work-related socioeconomic variables and maternal smoking larger studies are needed.

The high response rate in the current study (87%) reduced the risk of selection bias. However, we did not have information about the women who did not participate to perform a non-responder analysis. The prevalence of smoking in our study was similar to a crude estimate of pregnant smokers (6.7%) found in the Danish National Birth Registry (2016) [28]. Also, we compared the population in this study to the general pregnant population in Denmark and found that according to the variables available from the Danish National Birth Register from 2017 (BMI, parity, age, ART) the populations were comparable, except from a larger proportion of multiparas in our study population [28]. Non-Danish-speaking women were excluded, and the results can therefore only be generalized to the Danish-speaking part of the population.

It is well known that smoking is underestimated in self-reported data and studies have shown an underestimation of 9–11% compared to objective measurements [46]. Hence the prevalence of smoking in our study was potentially higher than reported. However, self-reported smoking behavior is often used in population-based studies as a valid indicator for tobacco exposure [46].

### Clinical implications

Our study confirms that social inequality in smoking still exists among pregnant women in a part of Denmark, at time of conception and during pregnancy. The World Health Organization (WHO) is working towards a “Smoke-free Denmark” in 2030 [47] which is part of a worldwide WHO initiative to eliminate the harmful effects of smoking. One of the strategies to reach this goal is to provide smoking cessation interventions to vulnerable groups, such as pregnant smokers and smokers with low socioeconomic status, to decrease the social inequality that has been shown in smoking patterns [35]. Our findings support the WHO strategy: interventions should be targeted toward the groups with low socioeconomic status, including multiparous women and women smoking ≥10 cigarettes daily.

Our findings also suggest that interventions should be intensified early in a woman’s first pregnancy or even before pregnancy when possible, as prior evidence suggests that life style changes and optimization of health in the preconception period, including smoking cessation, play a major role in the life course of health for mother and child [48]. Furthermore, in this present study as well as in previous nulliparous women are more likely to stop smoking [29, 34] and motivation for quitting has been found to be higher in the beginning of pregnancy [49]. Interventions should likewise aim to sustain smoking cessation in the post-partum period, as there is evidence showing that more than 40% relapse within six months after childbirth [50]. The prevalence of smoking relapse after childbirth is not known in the Danish population and needs to be investigated further.

## Conclusion

In conclusion, this study showed that the prevalence of smoking at the time of conception and at 20 weeks of gestation has decreased in this regional Danish population. We estimated the prevalence to be 16% at the time of conception and 6% at 20 weeks of gestation. At the time of conception, the three groups of smokers, quitters and non-smokers differed in maternal age, BMI, parity, education, employment status, work schedule and cohabitation. Predictors for maternal smoking were socioeconomic parameters indicating a continued social inequality in smoking during pregnancy.

## Data Availability

The datasets generated and/or analyzed during the current study are available in the Mendeley repository, doi:10.17632/zg2xn44k4c.1

## Abbreviations

aOR: Adjusted Odds Ratio
ART: Assisted Reproductive Technologies
BMI: Body Mass Index
CI: Confidence Interval
OR: Odds Ratio
